# Supplementary material for: Impact of odorants on perception of sweetness by honey bees
Source: PLoS One. 2023 Dec 27;18(12):e0290129. doi: 10.1371/journal.pone.0290129 (PMC10752549; doi:10.1371/journal.pone.0290129)
Supplement: Attachment — Submitted filename: Response to Reviewers Pel et al.doc [file pone.0290129.s002.doc]

November 13, 2023

To the Editor:

Thanks to the two reviewers and the Editor for helpful comments on our submitted article. These comments have led to revisions that we believe have improved our report and prepared it for publication. Here is our point-by-point response to the reviewers’ thoughtful comments.

**Response to Reviewer #1**

We agree that there is no reason to expect that the taste sensory system of humans and honey bees are the same. In fact, we avoided assertions of analogous structures and pathways and focused on the knowledge base of honey bees feeding on nectar in the field and on sucrose solutions in experimental situations. The study was primarily an exercise in curiosity based on the demonstrated relationship between plant odorants and sweetness perception in humans. It was inspired by a lively conversation at a conference with Professor Linda Bartoshuk (currently of the University of Florida Center for Smell and Taste) on the comparable phenomenon in humans [e.g., Bartoshuk LM & Klee HJ. (2013) Better fruits and vegetables through sensory analysis. *Current Biology* 23: R374-8.] She directed us to further literature in this area [e.g., Baldwin EA, Goodner K & Plotto A. (2008) Interaction of volatiles, sugars, and acids on perception of tomato aroma and flavor descriptors. *Journal of Food Science* 73:S294-307.] Subsequent discussions with sensory physiologists at our home institution of Wake Forest University (Wayne Silver, Joost Maier) encouraged us to conduct the experiments reported in the present article. The current project is empirical, not theoretical, although we believe it contributes to the understanding of the multiple determinants of food acceptance in honey bees. We agree that the results are perhaps not surprising but feel that we were careful not to overgeneralize results obtained in the laboratory to claim that floral odorants modify foraging behavior under natural conditions. The results are helpful to us in thinking and designing future experiments on the function of geraniol [(2E)-3,7-dimethyl-2,6-octadien-1-ol] as a signal in honey bee life. We think that these data might be helpful to other researchers interested in the odorant environment experienced by pollinators.

We have added a comment to the effect that this project was curiosity-driven to the Introduction (Lines 75-77). We do think, however, that it is an oversimplification to assert that all food selection decisions by honey bees are made at the colony level, given that a honey bee colony can exploit multiple food sources at the same time and that modeling suggests decentralized control of foraging decisions [Seeley TD, Camazine S, Sneyd J. (1991) Collective decision-making in honey bees: how colonies choose among nectar sources. *Behavioral Ecology & Sociobiology* 28:277-290.]We thank Reviewer #1 for the recommendation to shorten the Discussion and have made some cuts in that section (the previous conclusion was deleted; replaced by lines 379-386 which are now 471-478.

**Response to Reviewer #2**

Lines 51-56, also 58. This reviewer asked about the sugar composition of the different berries used in human studies. We are interested in this, too! We thought that the use of a single sugar (sucrose) in our studies would be a more rigorous test for odorant-induced changes in behavior but have of course wondered if there is an odorant x sugar interaction in both humans and honey bees. It was reported in a 2019 comparison of the sugar profiles of strawberries and blueberries that the three most abundant sugars in the tested cultivars of strawberries were glucose, fructose, and sucrose, while the three most abundant sugars in blueberries were glucose, fructose, and galactose [Akšić MF, Tosti T, Sredojević M, Milivojević J, Meland M, Natić M. (2019) Comparison of sugar profile between leaves and fruits of blueberry and strawberry cultivars grown in organic and integrated production system. *Plants* (Basel). 8: 205.] Although we chose not to explore a possible odorant x sugar interaction in our initial study, we now mention this information briefly in the Introduction (Lines 56 and 58-59).

Line 142: We agree that 2.9 mol L^-1^ is a high sucrose concentration. We wanted to use a high concentration that almost any honey bee will respond to ensure that each of our subjects, despite being manipulated for harnessing, were physically capable of responding. This is similar to the use of honey as a positive control in other studies of sucrose sensitivity in honey bees [e.g., Siegel AJ, Freedman C, Page RE Jr. (2012) Ovarian control of nectar collection in the honey bee (*Apis mellifera*). PLoS One 7:e33465.] We chose to use a highly concentrated sucrose solution for this purpose to avoid the variability inherent in honey. This is now discussed briefly in Lines 147-150, and the Siegel et al. (2012) reference has been added.

Line 161-170: The use of water between presentations of sucrose solution of different concentrations was to ensure that the honey bees responded selectively to the gustatory stimulus presented rather than to the mechanosensory stimulus of the light tap on the antenna or as a result of inadvertent learning by contact of the proboscis with the antennae. These data were not analyzed given the lack of any hypothesis regarding the effect of odorants on responses to water, but any subject that responded indiscriminately to every presentation (water and sucrose) was removed from the analysis. We have added a statement to this effect in the Methods. Studies of responses evoked by antennal stimulation vary in whether they include or do not include “blank” trials between tastant presentations [e.g., blanks included: Pankiw T, Page RE Jr. (1999) The effect of genotype, age, sex, and caste on response thresholds to sucrose and foraging behavior of honey bees (*Apis mellifera* L.). *Journal of Comparative Physiology A* 185:207-13; Moauro MA, Balbuena MS, Farina WM. (2018) Assessment of appetitive behavior in honey bee dance followers. *Frontiers in Behavioral* Neuroscience 12:74; Guiraud M, Hotier L, Giurfa M, de Brito Sanchez MG. (2018) Aversive gustatory learning and perception in honey bees. *Scientific Reports* 8:1343; Bennett MM, Cook CN, Smith BH, Lei H. (2021) Early olfactory, but not gustatory processing, is affected by the selection of heritable cognitive phenotypes in honey bee. *Journal of Comparative Physiology A* 207:17-26] versus blanks not included: Wang Y, Brent CS, Fennern E, Amdam GV. (2012) Gustatory perception and fat body energy metabolism are jointly affected by vitellogenin and juvenile hormone in honey bees. *PLoS Genetics.* 8(6):e1002779]. Citations supporting our use of blanks have been added to Lines 170-180.

Line 199. The type of feeder used in studies of free-flying honey bees is described in detail in a technical paper by co-author Van Nest [Van Nest BN, Moore D. (2018) How to train a honey bee. *Journal of Undergraduate Neuroscience Education* 17(1):T1-T11]. All wells are filled at the start of the observation period, with each well containing approximately 375 µl of sucrose solution. This citation, which includes technical details, was already included in the paper. We added the volume of sucrose solution in each well to Line 209.

Line 260-263: We recorded the ambient temperature on each outdoor testing day. During the period these experiments were conducted the temperature ranged between a low of 25˚C to a high of 28.9˚ C. Although it is formally possible that temperature significantly impacted viscosity and bee physiology, we believe that the effects are likely to be negligible given that, over this temperature range, the density of water changes from 0.996 g/cm^3^ to 0.997 g/cm^3^. It is known that temperatures of body compartments in flying honey bees change with ambient temperature [Roberts SP, Harrison JF. (1999) Mechanisms of thermal stability during flight in the honeybee *Apis mellifera*. *Journal of Experimental Biology* 202:1523-33] but the changes over the temperature range we tested in are likely to be small. We have added information about the test temperature to the manuscript (Lines 283-287).

Line 341. As suggested by Reviewer 2, we have added a brief description of the Low, Medium and High labels to the figure caption so that the reader does not need to refer back to the Methods section. This information has been added to Lines 357-359.

Lines 351- 354. We concur with Reviewer 2 that “micronutrients in nectar also greatly affect subjective evaluation of nectar, even when sugar concentrations are equal.” This is an interesting topic for further study.

Line 398. In reference to the oldest honey bees tested in our study (hive-reared 35-day-old adult workers), Reviewer 2 pointed us to an interesting paper on age-related differences in conditioning of the proboscis extension response has been studied some, for example: Laloi, D., Gallois, M., Roger, B., Pham-Delègue, M.H., (2001) Changes with age in olfactory conditioning performance of cage-rearedworker honey bees (*Apis mellifera*). *Apidologie* 32: 231-242. Although the oldest honey bees tested in Laloi et al. study were 20-day-old-adult workers, we were interested to learn that the only age-based differences were resistance to extinction (not pertinent to this study) and spontaneous responses to the presentation of odorants, which were significantly higher in the youngest honey bees tested and non-existent in 20-day-olds. This speaks to our speculation that spontaneous responses to odorants may change over the course of a worker’s life, and we have included the citation in the revised manuscript (Lines 418-419). We used the ”Articles Citing this Article” function at the [Apidologie.com](../../../../Apidologie.com) website but did not recover any additional pertinent articles.

Line 402. Reviewer 2 notes that most studies of sucrose sensitivity use lower concentrations. We have reported our test conditions accurately and consistently in this article and look forward to expanding the range of concentrations used in future studies.

Journal policy comment on “data not shown” (line 255). These observations were anecdotal and made as we tested the implementation of procedures described in Van Nest BN, Moore D. (2018) How to train a honey bee. *Journal of Undergraduate Neuroscience Education 17*(1):T1-T11. Because our goal was always to test drinking duration in the field using a low but acceptable concentration of sucrose (0.3 mol L^-1^, 10%) that had been previously used as an intermediate value by other investigators, particularly Mujagic and Erber (2009). We have revised the description (Lines 263-265) in the methods section to remove the inappropriate reference to unanalyzed pilot data not shown as we based our choice of sucrose concentration for these studies on the published literature. In addition, as requested, we have provided captions for the supporting information files and checked the references.

We thank the Reviewers for their work. It is our hope that this manuscript is now acceptable for publication in PLoS One.

Susan E. Fahrbach, Ph.D.

Reynolds Professor of Developmental Neuroscience

Professor of Biology

Wake Forest University

Winston-Salem, NC 27109 USA
